# Analysis of Environmental and Typhoon Effects on Modal Frequencies of a Power Transmission Tower

**DOI:** 10.3390/s20185169

**Published:** 2020-09-10

**Authors:** Ting-Yu Hsu, Cheng-Chin Chien, Shen-Yuan Shiao, Chun-Chung Chen, Kuo-Chun Chang

**Affiliations:** 1 Department of Civil and Construction Engineering, National Taiwan University of Science and Technology, Taipei 10607, Taiwan; M10505326@mail.ntust.edu.tw; 2 National Center for Research on Earthquake Engineering, Taipei 10607, Taiwan; shengyuan@narlabs.org.tw (S.-Y.S.); jingochen@narlabs.org.tw (C.-C.C.); 3 Department of Civil Engineering, National Taiwan University, Taipei 10607, Taiwan; ciekuo@ntu.edu.tw

**Keywords:** environmental effect, Typhoon Nesat, power transmission tower, modal frequency, temperature, wind, long-term monitoring

## Abstract

The structural health monitoring of power transmission towers (PTTs) has drawn increasing attention from researchers in recent years; however, no long-term monitoring of the dynamic parameters of PTTs has previously been reported in the literature. This study performed the long-term monitoring of an instrumented PTT. An automated subspace identification technique was used to extract the dynamic parameters of the PTT from ambient vibration measurements taken over approximately ten months in 2017. Ten target modal frequencies were selected to explore the effects of environmental factors, such as temperature and wind speed, as well as the root-mean-square (RMS) acceleration response of the PTT. Variations in the modal frequencies of approximately 2% to 8% were observed during the study period. In general, among the environmental factors, the temperature was found to be the primary cause of decreases in the modal frequencies, except in the case of some of the higher modes. Typhoon Nesat, which affected the PTT on July 29th, 2017, seems to have decreased the modal frequencies of the PTT, especially for the higher modes. This reduction in the modal frequencies seems to have lasted for approximately two and a half months, after which they recovered to their normal state, probably due to a seasonal cool down in temperature. The reduction percentages in the modal frequencies due to Typhoon Nesat were quantified as approximately −0.89% to −1.34% for the higher modes, but only −0.07% to −0.46% for the remaining lower modes. Although the unusual reductions in the modal frequencies are reported in this study, the reason for this phenomenon is not clear yet. Further studies would be required in the future in order to find the cause.

## 1. Introduction

The vibration-based damage detection technique is among the promising techniques employed in the field of structural health monitoring. Generally speaking, it assumes that damage will alter the stiffness of a structure, which in turn will alter the measured dynamic responses of the structure. Although many approaches for detecting damage to structures in controlled laboratory environments have been successfully developed, the performance of these approaches in the field remains uncertain and requires validation. One of the main issues affecting the application of such techniques in the field that must be addressed is the effects of varying environmental conditions [1,2,3]. Many studies have reported that the variations in structural features due to varying environmental conditions such as temperature, boundary conditions, loading conditions, humidity, and wind speed can be much larger than those caused by structural damage [4,5,6]. In order to accommodate such environmental effects, it is very important to examine the influences of varying environmental conditions to acquire insight into these critical and complicated phenomena.

The majority of previous studies that have investigated such environmental effects have focused on bridge structures [7,8,9,10]. Some, however, have focused on buildings [11,12,13,14], especially ancient towers [15,16,17], and wind turbines [18,19]. Numerous field test conducted in such studies of the effects of environmental factors on these structures have indicated that temperature is the major factor that affects the natural frequencies of these structures [5,6,8,13]. It has also been found that temperature may affect modal frequencies in different ways for different mode shape patterns, e.g., bending modes, torsional modes, and local modes [15,16], especially the higher modes [16]. Meanwhile, increases in vibration intensity have been reported to cause decreases in modal frequencies [12,17,20]. Using data for Canton Tower, Zhang et al. observed that modal frequencies have a slightly descending trend with increasing temperature, wind speed, and vibration amplitude [21].

As for power transmission towers (PTTs), only a few studies of such environmental effects have been conducted. Huang et al. [22] recently installed an acceleration sensor and a meteorological sensor on a transmission tower located on the Xi’an Polytechnic University campus in order to measure the vibration responses and environmental parameters of the tower over the course of one week. They observed that the modal frequencies decreased as the temperature increased, and found that this relationship was quite linear. In another recent study, Huang et al. [23] developed an on-line monitoring system to collect data regarding the stresses affecting the post elements of a PTT, as well as ambient temperature and wind speed data, but no dynamic vibration data was collected in that study. Moreover, no analytical results using long-term data measured by the system developed by Huang et al. have yet been published. Zhang et al. used finite element analysis to study the wind-induced coupling vibration effects of PTT-line systems, and they concluded that wind speed plays an important role in the tower-line coupling effect. After a lab test, Carvalho et al. [24] installed a monitoring system including an accelerometer, swing angle sensor, digital camera, solar radiation sensor, temperature sensor, 3D wind speed sensor, and atmospheric pressure sensor on a PTT. However, as with the system developed by Huang et al., no analytical results for the system developed by Carvalho et al. have yet been reported in the literature.

Note that although a PTT is connected to other PTTs via cables, the cables are hanging on the PTTs via insulators. In normal conditions, little horizontal stiffness is contributed by the hanging cables. In addition, no sensors are allowed to be installed on the cables due to safety concerns. Therefore, there is no measurement system covering both towers and cables or two towers in the literature. Based on the literature review above, it seems reasonable to conclude that although the research community has started to pay greater attention to the long-term monitoring of PTTs, the effects of environmental factors on the properties of PTT structures have yet to be adequately investigated.

Because the manipulation of the excitation forces exerted on PTTs is not feasible due to the dangerous high-voltage electricity involved and various other practical issues, only output-only modal analysis (OMA) using ambient vibration measurements is suitable for identifying the modal parameters of PTTs. Stochastic subspace identification (SSI) is one of the most popular techniques of OMA, and has been verified as one of the techniques that provides the most accurate identification of natural frequencies and mode shapes even with high noise levels [25]. The SSI algorithm uses the projections of the future response onto the previous input/output response [26], and is very useful for modal identification of output-only systems subject to ambient vibration [27]. For the long-term monitoring of a PTT, large amounts of data need to be analyzed. If a high number of user interactions are required during the analysis process, then relatively large analysis times and costs will be required. Hence, the structural health monitoring of PTTs could be rendered impractical since such monitoring generally requires large amounts of data need to be processed in a short amount of time. As a result, automated modal parameter estimation is preferred for long-term monitoring. In this study, the fully automated, three-stage clustering approach for OMA developed by Reynders et al. [28] was employed. Using this automated OMA technique and no user interactions, this study sought to identify the modal parameters of a PTT installed with vibration sensors and meteorological sensors for long-term monitoring. Through the long-term monitoring data, which included data for one typhoon event, this study investigated the effects of the environment in general and the typhoon in particular on the identified modal frequencies.

## 2. Investigated Power Transmission Tower and Its Monitoring System

**Figure 1:**
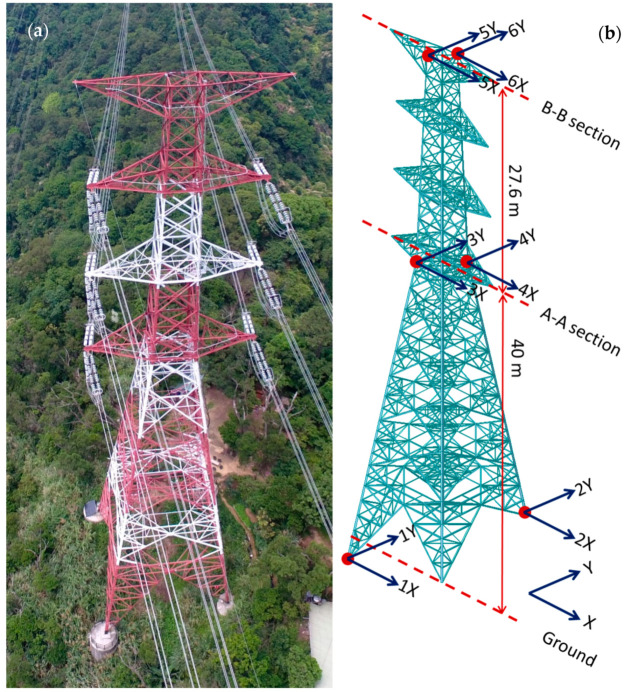
(**a**) Overview of the PPT; (**b**) Locations and Directions of the accelerometers.

**Figure 2:**
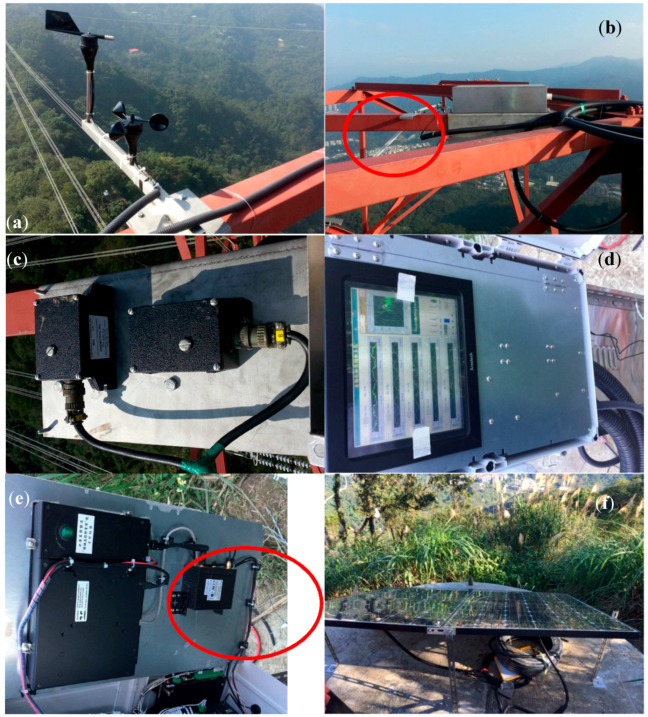
The monitoring system. (**a**) wind speed and wind direction sensors; (**b**) temperature sensor mounted on the component of PTT; (**c**) accelerometers; (**d**) data acquisition system with a monitor; (**e**) 4G wireless router; (**f**) system power set.

The investigated PTT structure was a G4-345 kV strain tower (Figure 1a). Because the PTT structure is located on the slope of a hill in New Taipei City, Taiwan, the lengths of its legs are different. Unlike bridges and buildings, there are almost no human activities occurring on the PTT under normal operational conditions. Moreover, it is located in an isolated area; hence, even human activities in the surrounding area are rare. The major excitation source affecting the PTT is probably the wind. One temperature sensor, one wind speed sensor, and one wind direction sensor were installed on the top of the PTT, as shown in Figure 2a,b. In addition, two forced-balanced accelerometers (Kinemetrics FBA-11) for each horizontal direction were installed in two positions at each of three different elevations. As such, a total of 12 degree of freedoms (DOFs) of acceleration were measured. The typical installation of the accelerometers at one of the points at the top elevation is shown in Figure 2c. The noise level of the accelerometer was approximately 0.0025 m/s^2^. The locations and directions of these sensors are also marked in Figure 1b. The Y-direction is longitudinal direction (parallel to the cables) while the X-direction is transverse direction. Note that although the accelerometers are mounted only on the tower, the vibration signals due to interaction between the tower and the cables can still be measured. If there is any vibration signal of the whole system is measured, the identified modal frequencies will reflect the behaviour of the system.

**Figure 3:**
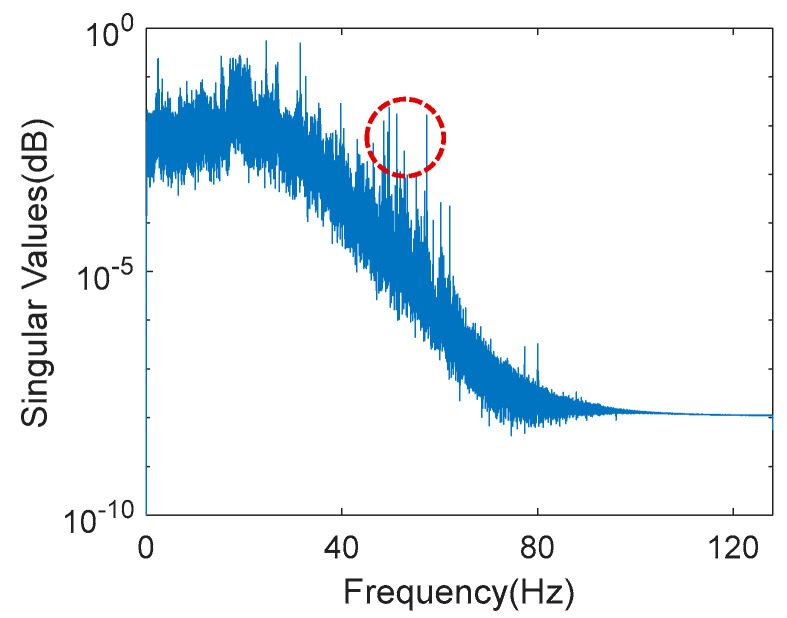
The singular value plot of the frequency domain decomposition of all the channels of a typical acceleration record.

The data acquisition system used was a 16-bit system, as shown in Figure 2d. The data was transmitted via 4G high-speed wireless networks, as shown in Figure 2e. The entire monitoring system was powered by a system power set comprising batteries and solar panels, as shown in Figure 2f. Since no powered network connection was available, in order to minimize the power consumption of the monitoring system, the acceleration signals were recorded only during the first ten minutes of each hour with a 256 Hz sampling rate, while the maximum wind speed and temperature measurements during the same ten minutes were recorded every hour. Figure 3 shows the singular value plot of the frequency domain decomposition of all the channels of a typical acceleration record when the tower was subjected to normal wind speed. The electric noise at approximately 50–60 Hz is indicated by the circle. Besides, it is also evident there is little content of data with a frequency higher than 40 Hz. Therefore, the original signals were resampled to 80 Hz and a 11th order of Butterworth filter with 32 Hz cut-off frequency was used to prevent aliasing effect after resampling. The operation of the monitoring system was started on 8 March, 2017, and the data collected by the system from then until 31 December, 2017, were used in this study. It should be noted, however, that the collection of data was interrupted during several periods due to technical problems such as low battery power and the system being shut down. The longest period of interruption was from 11 June through 5 July.

## 3. Automated Modal Analysis

In this section, the SSI algorithm and the automated modal analysis procedure modified from Reynders et al. [28] is summarized, with the dataset measured at 13:00 on April 12th being adopted herein as a typical example.

### 3.1. SSI

The SSI is derived based on the state space description of a linear dynamic system. Assuming the vector of output measurements at the k-th time interval,$y_{k}$, with r time samples being available, the Hankel matrix can be constructed as follows:

(1) $$H^{}\equiv \left[\frac{\begin{array}{cccc} y_{0}^{} & y_{1}^{} & \cdots & y_{j-1}^{}\\ y_{1}^{} & y_{2}^{} & \cdots & y_{j}^{}\\ \cdots & \cdots & \cdots & \cdots \\ y_{i-1}^{} & y_{i}^{} & \cdots & y_{i+j-2}^{} \end{array}}{\begin{array}{cccc} y_{i}^{} & y_{i+1}^{} & \cdots & y_{i+j-1}^{}\\ y_{i+1}^{} & y_{i+2}^{} & \cdots & y_{i+j}^{}\\ \cdots & \cdots & \cdots & \cdots \\ y_{2i-1}^{} & y_{2i}^{} & \cdots & y_{2i+j-2}^{} \end{array}}\right]\equiv \left[\frac{Y_{p}^{}}{Y_{f}^{}}\right]\in {\mathbb{R}}^{2li\times j}$$

where i is a user-defined index and must be larger than the order n of the system. The Hankel matrix is divided into the past, $Y_{p}\in {\mathbb{R}}^{li\times j}$, and the future, $Y_{f}\in {\mathbb{R}}^{li\times j}$, parts, where j equals r−2i+1. Then the orthogonal projection of the row space of the matrix $Y_{p}$ on the row space of the matrix $Y_{f}$ is defined as $O_{i}^{t}$, which can be calculated by the following formula:

(2) $$O_{i}^{t}=Y_{f}/Y_{p}\equiv Y_{f}Y_{p}{}^{T}{\left(Y_{p}Y_{p}{}^{T}\right)}^{†}Y_{p}\in {\mathbb{R}}^{li\times j}$$

where / denotes the projection operator, ${}^{T}$ denotes the transpose operator, and ${}^{†}$ denotes the pseudo-inverse operator. Singular value decomposition of the orthogonal projection $O_{i}^{t}$ is performed, and the singular values are split into two parts as follows:

(3) $$O_{i}^{t}=USV^{T}=\left(\begin{array}{cc} U_{1} & U_{2} \end{array}\right)\left(\begin{array}{cc} S_{1} & 0\\ 0 & S_{2} \end{array}\right)\left(\begin{array}{c} V_{1}^{T}\\ V_{2}^{T} \end{array}\right)\approx U_{1}S_{1}V_{1}^{T}$$

in which $S_{1}$ contains the first n singular values. The extended observability matrix can be obtained by:

(4) $$\Gamma_{i}=U_{1}S_{1}^{1/2}\equiv \left[\begin{array}{c} C\\ CA\\ \vdots \\ CA^{i-1} \end{array}\right]\in {\mathbb{R}}^{li\times n}$$

Then the system parameter matrix A can be determined as follows:

(5) $$A={\underline{\Gamma }}_{i}{}^{†}{\bar{\Gamma }}_{i}$$

Using the eigenvalues and eigenvector of the system parameter matrix A as well as the output matrix C, the undamped modal frequency $f_{i}$, damping ratio $\xi_{i}$, and modal vector $\varphi_{i}$ can be calculated according to the theory of linear systems. The analysis program of SSI was coded using Matlab software, and the results using the SSI had been checked against the MACEC toolbox developed at the Structural Mechanics Section of KU Leuven [29].

**Figure 4:**
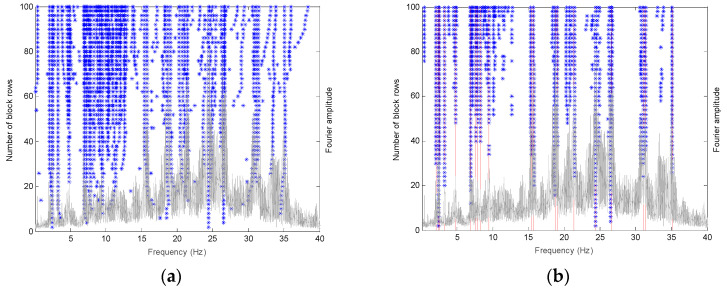
A typical stabilization diagram of the dataset measured at 13:00 of 12 April (**a**) before and (**b**) after it was cleared with the selected modes marked with vertical red lines.

Conventionally, in order to determine the modal parameters using the SSI algorithm, the stabilization diagram is usually constructed to detect the representative stable modes. The user-defined index i was fixed at 70, hence the dimension of H equals 1680 × 23862, which should be sufficient to identify the physical modes of the PTT. The stabilization diagrams were constructed with the number of singular values n ranging from 2 to 100. The typical stabilization diagram of the PTT of the dataset measured at 13:00 on April 12th is shown in Figure 4a. It can be observed that there are some modes in the figure that are not stable or representative enough.

### 3.2. Automated Modal Clustering Algorithm

Because investigating a stabilization diagram to determine the representative stable modes is subjective and time consuming, especially for long-term monitoring with continuous large amounts of data, we adopted the three stages clustering algorithm proposed by Reynders et al. (2012) to automatically detect the representative stable modes in this study.

**Figure 5:**
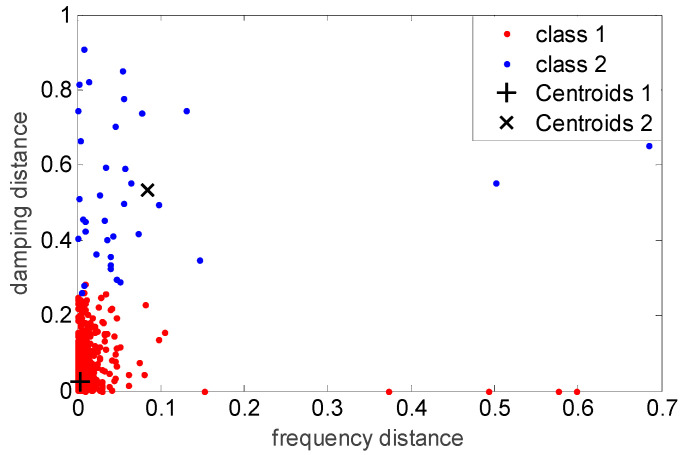
The distribution of the two clusters of the dataset measured at 13:00 of 12 April in the two-dimensional projections of frequency distance vs. damping distance.

The first stage consists of the automated clearing of a stabilization diagram using single-mode validation criteria. In this stage, the single-mode validation criteria are calculated, and then the modes are classified as certainly spurious or possibly physical using a 2-means clustering algorithm. The single-mode validation criteria employed in this study included the distance measures of eigenfrequency, the distance measures of eigenfrequency damping ratio, the modal assurance criterion (MAC), the modal phase collinearity (MPC), and the mean phase deviation (MPD) [28]. The modes were then represented by a 5-dimensional vector containing these five validation criteria. The 2-means clustering algorithm was applied using these 5-dimensional vectors, denoted as $p_{j}$. It was used to minimize the sum of the squared Euclidian distances between $p_{j}$ and the nearest cluster centroid $p_{ck}$. Here, $p_{c1}$ denotes the centroid of the cluster of possibly physical modes, while $p_{c2}$ denotes the centroid of the cluster of certainly spurious modes, and they are calculated as follows:

(6) $$\left\{p_{c1},p_{c2}\right\}=\underset{p_{ck}}{\arg s\min}\sum_{k=1}^{2}\sum_{j=1}^{n_{m}(k)}{\left\|p_{j,c}-p_{ck}\right\|}_{2}^{2}$$

where $n_{m}(k)$ denotes the number of modes in each cluster k. When the distance of the mode is closer to $p_{c1}$, i.e., when $\left\|p_{j}-p_{c1}\right\|\leq \left\|p_{j}-p_{c2}\right\|$, it becomes the members of the $p_{c1}$ set. Otherwise, it becomes the members of the $p_{c2}$ set. The objective function in Equation (6) is minimized in an iterative optimization process. The details of this iteration process can be found in [28]. After the convergence of the clustering process, the modes classified as certainly spurious are then removed from the stabilization diagram. The distribution of the two clusters of the dataset measured at 13:00 on April 12th in a typical two-dimensional projection of the five-dimensional space of the single-mode validation criteria is shown in Figure 5. Finally, since the damping ratio of a steel structure should be quite small, e.g., approximately 2%, and should not be negative, the modes with a damping ratio larger than 10% or smaller than 0% were also removed.

**Figure 6:**
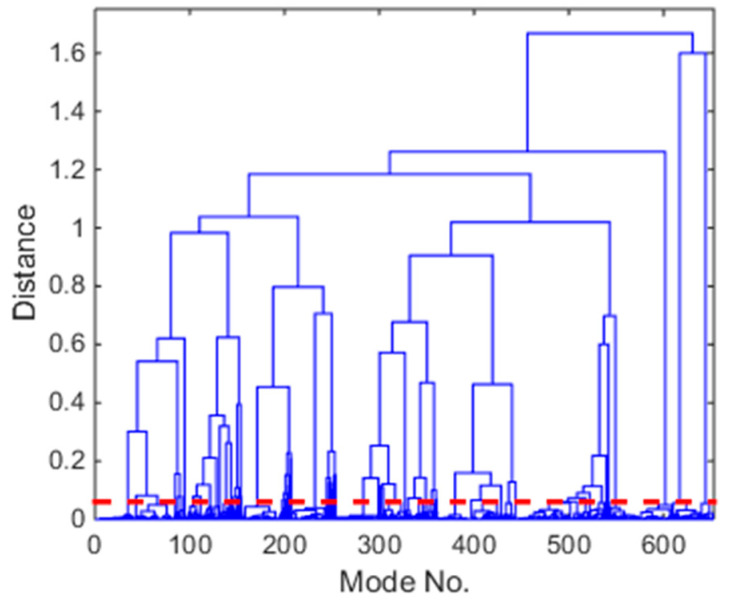
Dendrogram of the hierarchical clustering step (full lines), and automatically determined cut-off distance (bold dashed line).

After the first stage, the stabilization diagram has been cleared, as shown in Figure 4b, but there are still some spurious modes. In the second stage, the similar modes in the cleared stabilization diagram are grouped together using a hierarchical clustering approach. This stage serves as a visual inspection of the stabilization diagram to find vertical lines of stable modes, but in an automated way. Initially, all of the modes are considered as a single cluster, and then the distance between any two clusters k and l is calculated using the following equation:

(7) $$d(k,l)=d(\lambda_{k},\lambda_{l})+1-MAC(\phi_{k},\phi_{l})$$

where λ represents the eigenvalue and ϕ represents the mode shape. Then, the closest two clusters are collected in a single cluster, and the distance between all the clusters is updated as the average distance between their elements. This updating of distance will repeat until the distance exceeds a threshold calculated as follows:

(8) $$\tilde{d}=\mu_{p1}+2\sigma_{p1}$$

where $\mu_{p1}$ and $\sigma_{p1}$ are the sample mean and sample standard deviation of all the distances calculated in the hierarchical clustering tree. The 652 possibly physical modes in the cleared stabilization diagram of Figure 4b are visualized as a hierarchical tree in Figure 6. It consists of П-shaped lines that connect the clusters in a hierarchical way, with the height of each П representing the distance between the two clusters being connected. The threshold of the distance, shown in Figure 6, yields 71 sets of similar mode sets from the cleared stabilization diagram.

Finally, we still need to determine which modes are stable and representative. The last stage of the process divides the 71 sets of similar modes into two clusters. It is expected that the sets of physical modes should contain many similar modes, while the sets of spurious modes should not. Here, the 2-means clustering algorithm is employed again to classify these two sets of modes. Since after the second stage, the number of sets of the physical modes should be much larger than the number of sets of the spurious modes, it is suggested that an additional number of empty sets equal to the number of mode sets containing more than one fifth of the number of modes in the largest set should be added [28]. Hence, there are $n_{h}$ mode sets in total, some of which are empty. Then, an iteration process similar to that used in the first stage is conducted again to automatically determine the physical mode sets and spurious ones via the updating of the centroids of the two clusters. Finally, using the typical dataset measured at 13:00 on April 12th, 20 physical mode sets were identified with the automated partitioning approach, as shown in Figure 4b, which is marked with vertical red lines. For each mode set, the mode with the median modal frequency value is selected as the representative one. Note that different modes could be identified using different datasets due to different environmental and excitation conditions.

A typical stabilization diagram of the dataset measured at 13:00 of 12 April (a) before and (b) after it was cleared with the selected modes marked with vertical red lines.

## 4. Continuous Monitoring of Modal Frequencies

Although approximately 20 modes could be identified with the automated modal analysis, unfortunately, due to varying environmental and excitation conditions, as well as the complexity of the PTT structure, these modes could not be successfully identified for every dataset. Hence, we needed to select the target modes for the investigation of the environmental and typhoon effects on these modes. In the current section, the procedure used to select the target modes to be investigated will be explained.

First of all, when the wind force is low, the vibration of the PTT is quite small, resulting in a signal-to-noise ratio that is also quite small. The quality of the identified modes would be quite poor using such a dataset. The root-mean-square (RMS) value of the 10-min acceleration measurements in two directions of one of the accelerometers in the B-B section (Figure 1b) is calculated to serve as an indication of the structural acceleration response level. The datasets with RMS acceleration values smaller than 0.006 m/s are excluded, and as a result, a total of 4647 datasets are available.

**Figure 7:**
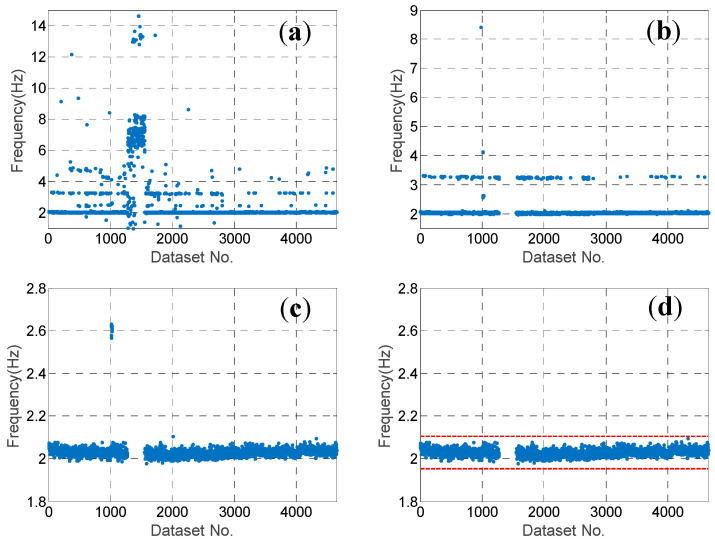
The results of the candidate mode of modal frequency 2.03 Hz at each step: (**a**) after the 1st step with the largest similarity coefficient in every dataset selected; (**b**) after the 2nd step with the ones with MAC≥0.8 left; (**c**) after the 3rd step with the MAC fulfilling the Tukey’s fence criterion; and (**d**) after the 4th step with the modal frequency fulfilling the Tukey’s fence criterion.

**Figure 8:**
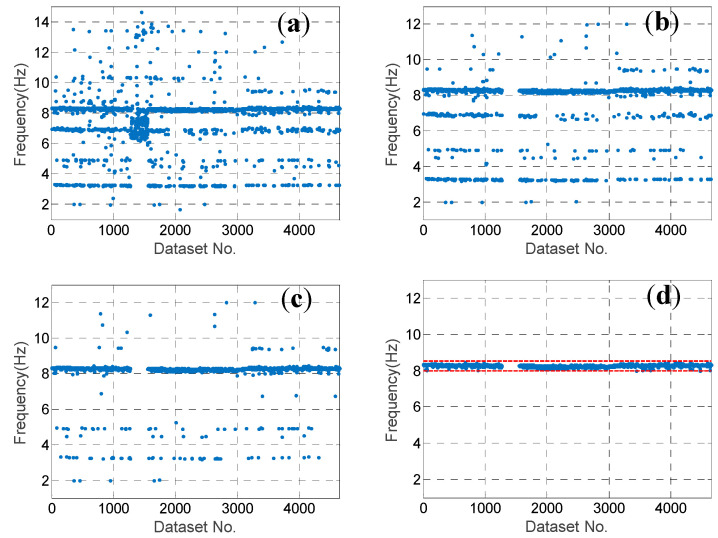
The results of the candidate mode of modal frequency 8.26 Hz at each step: (**a**) after the 1st step with the largest similarity coefficient in every dataset selected; (**b**) after the 2nd step with the ones with MAC≥0.8 left; (**c**) after the 3rd step with the MAC fulfilling the Tukey’s fence criterion; and (**d**) after the 4th step with the modal frequency fulfilling the Tukey’s fence criterion.

In order to track the identified modes automatically, we developed a procedure to screen out a candidate mode from all of the identified modes through all of the datasets. The candidate modes with relatively high identifiability were designated as the target modes to be studied in order to understand the environmental and typhoon effects. This process contained four steps. In the first step, a target mode with modal frequency $f_{c}$ and mode shape $\phi_{c}$ identified using one typical dataset were selected. The similarity coefficient between the candidate mode and the mode identified using the other dataset is defined as follows:

(9) $$\kappa_{e}=\frac{\sqrt{w_{f}\times D_{f}{}^{2}+w_{MAC}\times {\left(1-MAC\right)}^{2}}}{\sqrt{w_{f}+w_{MAC}}}$$

where

(10) $$D_{f}=\left|\frac{f_{i}-f_{c}}{f_{c}}\right|$$

represents the absolute difference ratio of two modal frequencies; $f_{i}$ represents the *i*th mode out of N modes identified using the other dataset; MAC represents the similarity between the two modes; and $w_{f}$ and $w_{MAC}$ represent the weighting coefficient of $D_{f}$ and (1-MAC), respectively. In this study, $w_{f}$ and $w_{MAC}$ are designated as 0.4 and 0.6, respectively. Note that the number of modes N identified using different datasets could be different, although the number was approximately 20 for most of the datasets. For each candidate mode, the mode with the largest similarity coefficient in every dataset was selected. The typical results of two modes, i.e., 2.03 Hz and 8.26 Hz, are illustrated in Figure 7a and Figure 8a, respectively. Note that for many datasets, the selected mode with the largest similarity coefficient could evidently be the wrong one. This is because for these datasets, the candidate mode is not identified.

**Figure 9:**
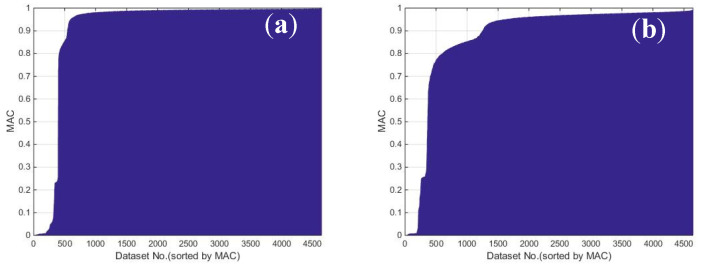
The sorted MAC values of the two typical candidate modes: (**a**) modal frequency 2.03 Hz; and (**b**) modal frequency 8.26 Hz.

In order to eliminate the wrong modes from among the selected ones, three more steps were employed. In the second step, the MAC values between the candidate mode and the selected modes were investigated. The sorted MAC values of the two typical candidate modes are shown in Figure 9. It can be observed that there was a platform with a MAC value larger than 0.85, which means that for most of the selected modes, the MAC value was larger than 0.85. Hence, we eliminated the selected modes with MAC values smaller than 0.85, and the typical results of two modes after the second step are shown in Figure 7b and Figure 8b.

In the third step, the threshold of the MAC value was further designated according to the Tukey’s fence criterion [30] in order to eliminate the outliers. The $M_{Q1}$ and $M_{Q3}$ are the lower and upper quartiles of the MAC values, respectively, when using the selected modes with MAC≥0.85. Because the MAC values should be close to 1.0, only the lower threshold $M_{th}$ is designated as follows:

(11) $$M_{th}=M_{Q1}-k(M_{Q3}-M_{Q1})$$

for some nonnegative constant k{\displaystyle k}. It is suggested that k=3 should be used to indicate that these modes are “far out”. The typical results of two close modes after the third step are shown in Figure 7c and Figure 8c. Evidently, there were still some selected modes that were wrong, with modal frequencies far from those of the candidate modes. This is because the number of measured DOFs of the mode shape was too few given the complexity of the PTT structure. Hence, it is not easy to distinguish the modes using only the MAC value.

In the fourth step, the threshold of the modal frequency was designated according to the Tukey’s fence criterion again in order to eliminate the wrong modes with relatively high MAC values. Both the upper and lower thresholds were designated as follows:

(12) $$F_{th}=[F_{Q1}-k(F_{Q3}-F_{Q1}),F_{Q3}+k(F_{Q3}-F_{Q1})]$$

where $F_{Q1}$ and $F_{Q3}$ are the lower and upper quartiles of the modal frequency, respectively, using the selected modes processed after using the threshold $M_{th}$ in the third step. The typical results are illustrated in Figure 7d and Figure 8d. Obviously, only the wrong modes have been removed.

**Figure 10:**
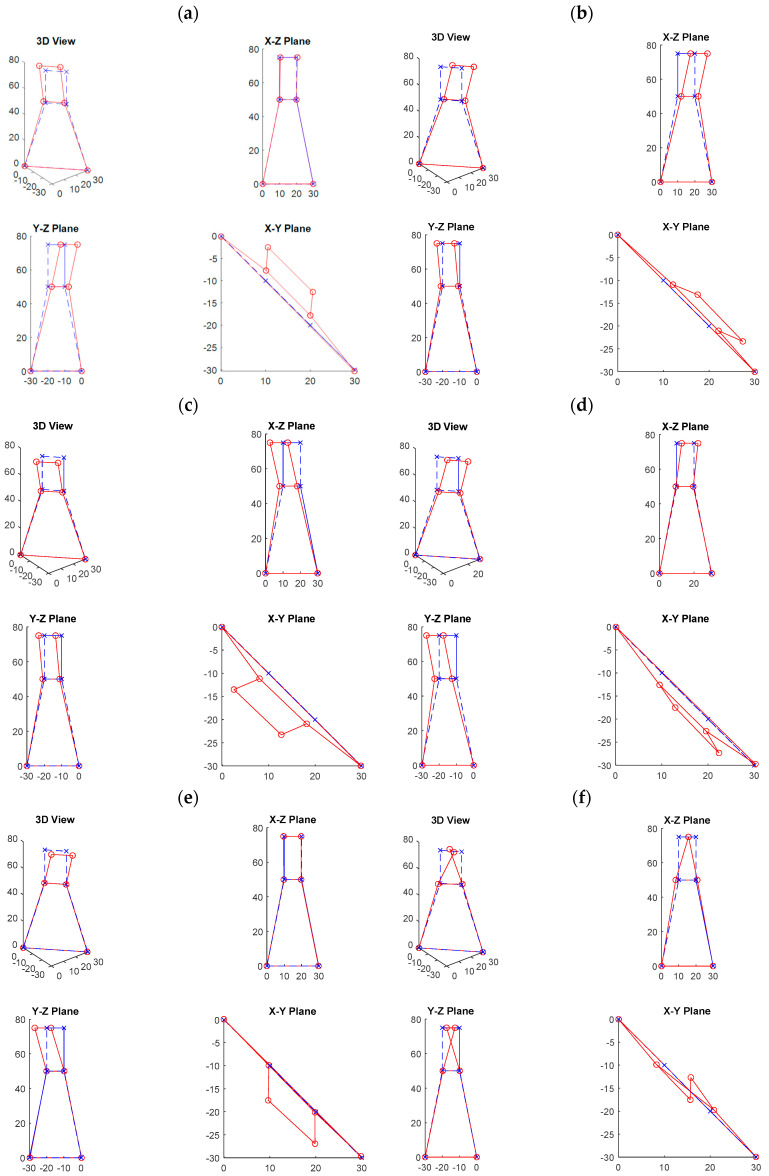

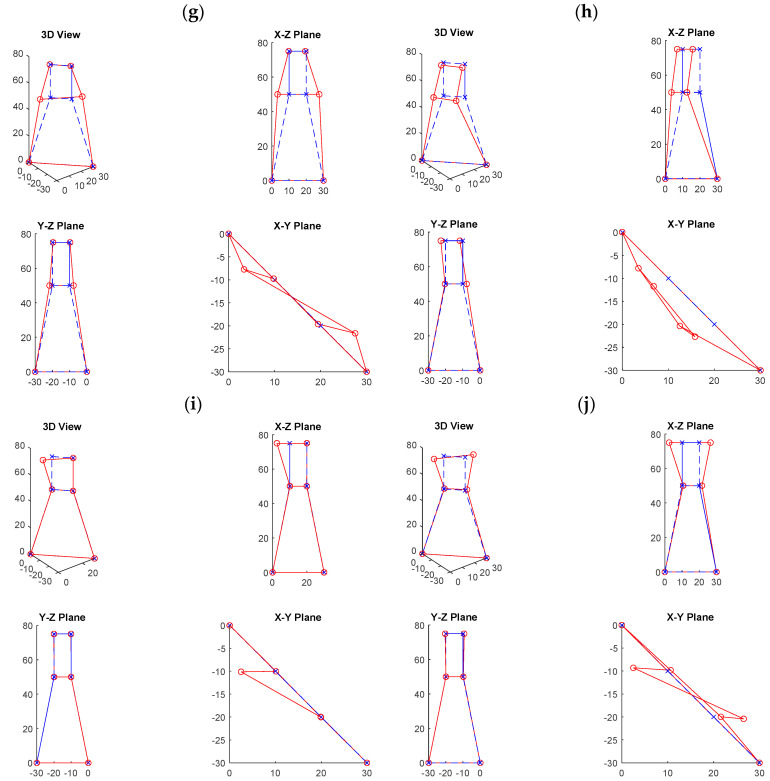
The mode shapes of the ten target modes with modal frequency: (**a**) 2.03 Hz; (**b**) 2.43 Hz; (**c**) 2.51 Hz; (**d**) 3.26 Hz; (**e**) 8.26 Hz; (**f**) 15.33 Hz; (**g**) 18.67 Hz; (**h**) 21.39 Hz; (**i**) 24.36 Hz; and (**j**) 31.08 Hz.

After using the above four-step procedure, ten candidate modes with identifiability larger than 60% were designated as the target modes, as listed in Table 1, and these modes were used to investigate the environmental and typhoon effects on the modal frequencies. The identifiability values of the first four modes were much higher, at approximately 95% and higher, while those of the six higher modes were much lower, ranging from approximately 60% to 80%. The mode shapes of these target modes are shown in Figure 10. Because the number of measured DOFs of the mode shape was too few given the complexity of the PTT structure, it was not easy to investigate the mode shapes and draw conclusions regarding the pattern of the shapes. Nevertheless, we still separated the modes into three categories, as listed in Table 1. The first category consisted of the modes that appeared to be “bending” along the vertical axis. These modes were modes No. 1, 2, 3, 4, 5, and 8. Three modes belonged to the “torsion” category, the second category. They were modes No. 6, 7, and 10. Only mode No. 9 seemed to behave differently, where only the amplitude of one DOF was larger. We thus concluded that this mode belonged to the “local” category.

## 5. Analysis of Environmental and Typhoon Effects

In this section, we describe the analysis of the long-term monitoring data of the PTT undertaken to investigate the trends in the variations of the identified target modal frequencies and better understand the effects from the environmental factors. Besides the temperature and wind speed, the acceleration response level of the PTT was also considered in this study because several previous works in the literature [12,20] have revealed that it may remarkably alter the modal frequencies of civil structures. Hence, the RMS value of the acceleration measurements in two directions of one of the accelerometers in the B-B section was calculated and served as an indication of the structural acceleration response level.

**Figure 11:**
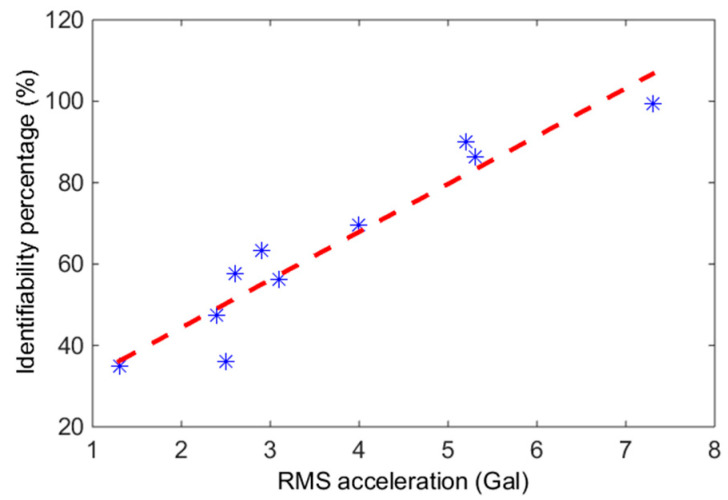
The relationship between the average RMS acceleration value and the identifiability percentage of each month with the blue stars representing data and the red dashed line representing the simple linear regression result.

First of all, we performed a statistical analysis to investigate the percentage of identifiability for each identified modal frequency. Notwithstanding some periods during which vibration signal data were not collected due to technical problems, in total, there were 4793 available acceleration datasets with a length of 10 min. The percentage of identified modal frequencies $\varepsilon_{j}$ is defined as the ratio between the total number of the j-th identified target modal frequencies and the total number of measured acceleration datasets (one dataset per hour). We found that the percentage of identifiability of each identified modal frequency of each month was highly correlated to the average level of excitation. That is, the higher the average RMS acceleration value for each month, the higher the identifiability percentage was, as shown in Figure 11. The Pearson correlation coefficient between the average percentage of all the identified modal frequencies and the average RMS acceleration value of each month was 0.95, which is quite high. This result makes sense, however, since when the amplitude of the vibration signal is small, the signal-to-noise ratio of the measured acceleration time history is low, and then the quality of the identified modal parameters is not good.

**Figure 12:**
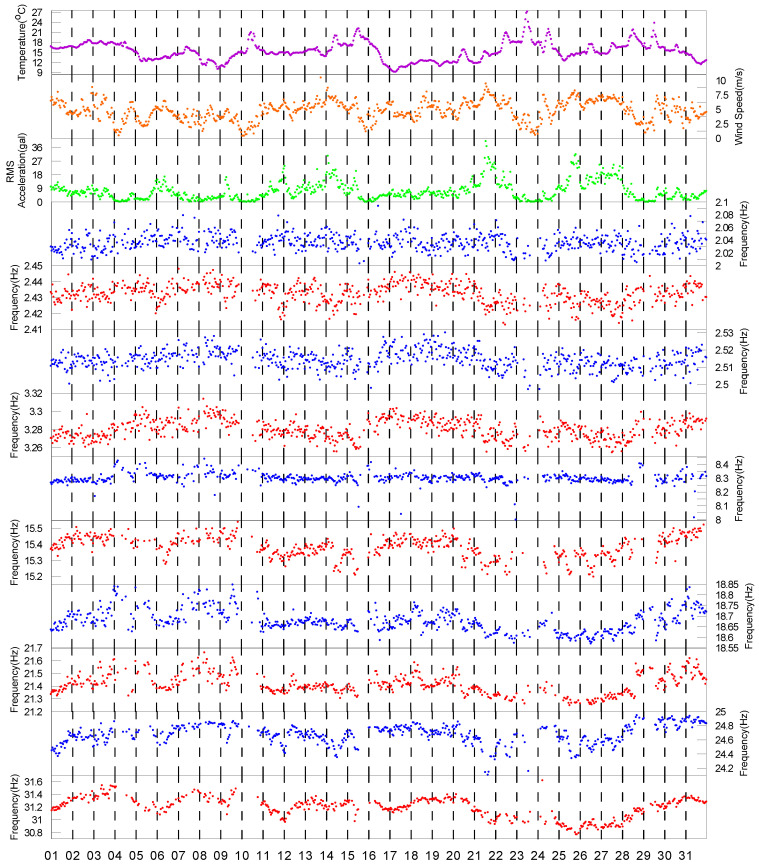
Comparison of identified modal frequencies while considering three different environmental factors in December of 2017.

In order to observe the relationship between the target modal frequencies and the corresponding variations of the three considered environmental factors, the data for the month with the highest percentage of identified modal frequencies, i.e., December, were selected, and are shown in Figure 12. Unfortunately, it is not easy to understand the environmental effects on the modal frequencies by referring to Figure 12.

**Figure 13:**
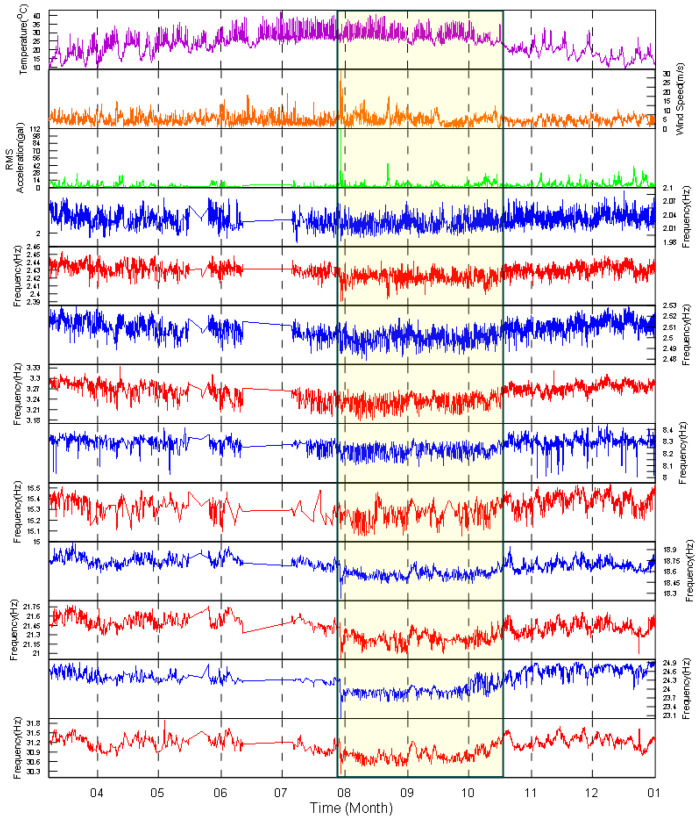
Comparison of identified modal frequencies while considering three different environmental factors in 2017. The middle section with a yellow background represents the phase II.

Since it is not easy to understand the environmental effects on the identified modal frequencies via the data for only one month, we stepped back to observe the data collected in 2017 over approximately ten months, as shown in Figure 13. First, the range of variation in the modal frequencies during the whole year caused by the environmental factors was studied. The average variation range, i.e., the maximum minus the minimum values, and the variation percentage, i.e., the range of variation divided by the average value, were calculated and are listed in Table 1. Overall, the variation range for all the modes was below 8%. The largest variation range was the range for the 9th mode, which was approximately 7.9%, while the smallest two ranges were those for the 2nd mode and the 3rd mode, which were approximately 2.2% and 2.6%, respectively. The variation range of the target modal frequencies of the TPP is thus not as high as those for bridges and buildings, for which the variation range can be approximately 10% to 20%, as shown in [5,12].

**Figure 14:**
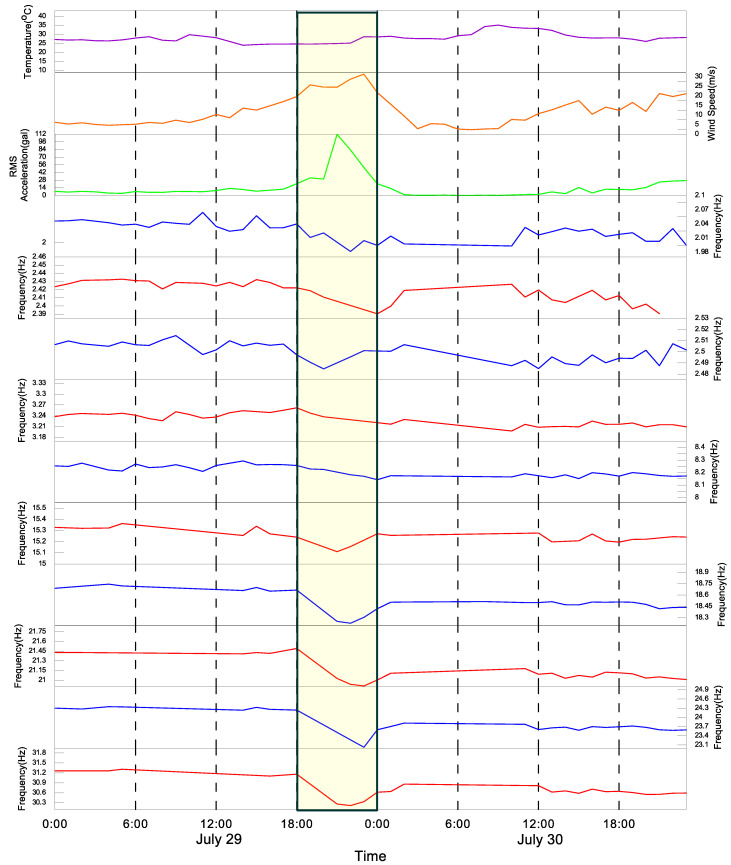
Comparison of identified modal frequencies while considering three different environmental factors on July 29th and 30th. The middle section with a yellow background represents the period with wind speed exceeding 17.2 m/s.

During the long-term monitoring of the PTT conducted in 2017, Typhoon Nesat, which injured 111 people and caused US$5.83 million of agricultural losses in Taiwan, affected the PTT on July 29th. In order to observe the effect of the typhoon and other environmental factors on the modal frequencies, the close view during the typhoon period in plotted in Figure 14. As can be observed clearly in the figure, during the hit of Typhoon Nesat, the wind speed measured on the PTT exceeded the 17.2 m/s (the minimum wind speed to be classified as a typhoon) for seven hours. The maximum wind speed was approximately 32 m/s, which induced the maximum RMS acceleration value of 1.11 m/s^2^ at 21:00 UTC+8. No visual damage was observable either on the PTT or the cables after the Typhoon Nesat event. The power transmitting system kept in normal operation condition without any interference due to this typhoon event. No abnormal behaviour was observed after examining the measured environmental and acceleration signals during the typhoon event. However, it was found that the modal frequencies of some of the higher modes, i.e., the 7th mode to the 10th mode, dropped substantially then, after which their frequencies remained lower until the middle of October when the temperature started to cool down. However, this phenomenon could not be clearly observed for the rest of the modes, especially the lower modes.

In order to understand the effects on the modal frequencies caused by the typhoon event and the long-term environmental variation, we divided the data collected in 2017 into three phases, as marked in Figure 13. The first phase, which started on March 8th and ended at 20:00 on 29 July, served as the phase of data before the Typhoon Nesat event; the second phase, which started at 21:00 on 29 July and ended on 14 October, served as the phase of data after the Typhoon Nesat event; and the third phase, which started on 15 October and ended on 31 December, served as the phase of data for the recovery from the Typhoon Nesat event.

Firstly, we calculated the Pearson correlation coefficients between the three environmental factors and the modal frequencies during the first phase, as shown in Table 2. In general, compared to the other two factors, the temperature affected the modal frequencies to a greater extent, with the exception of the 7th mode and the 8th mode where the RMS acceleration values had the highest correlation coefficients. Among all the temperature correlation coefficients, the two modes with the highest correlation coefficients were the 4th mode and the 3rd mode, and their correlation coefficients were −0.90 and −0.77, respectively. For the wind speed, all the correlation coefficients were negative, and their values were between −0.08 and −0.40, which are not high. As for the RMS acceleration, the correlation coefficients were between −0.41 and 0.34. Note that for the first five modes, the RMS acceleration correlation coefficients were positive, and for the remaining higher modes, the RMS acceleration correlation coefficients were negative. It seems that the modal frequencies also slightly decreased with the increases in RMS acceleration values, but only for the higher modes.

**Figure 15:**
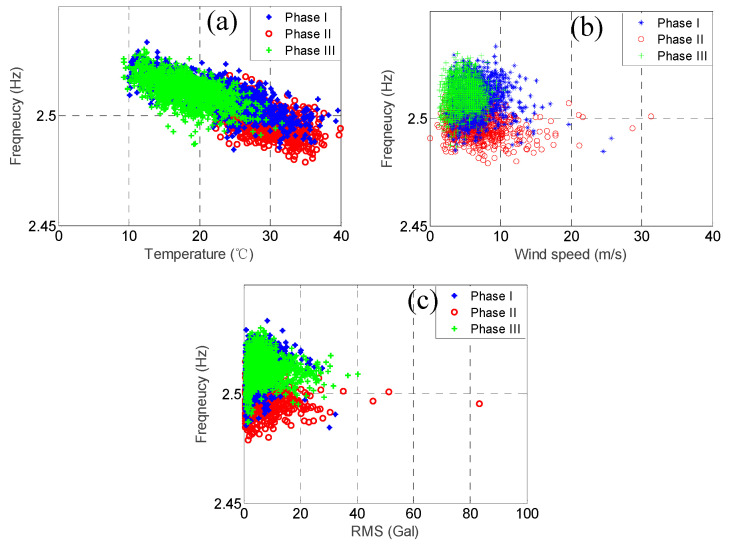
The relationship between the 3rd modal frequency (2.51 Hz) and (**a**) temperature; (**b**) wind speed; and (**c**) RMS acceleration.

**Figure 16:**
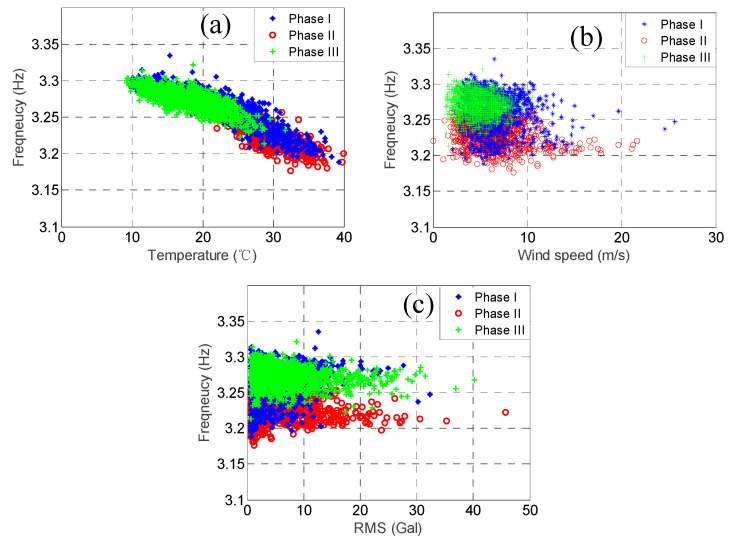
The relationship between the 4th modal frequency (3.26 Hz) and (**a**) temperature; (**b**) wind speed; and (**c**) RMS acceleration.

**Figure 17:**
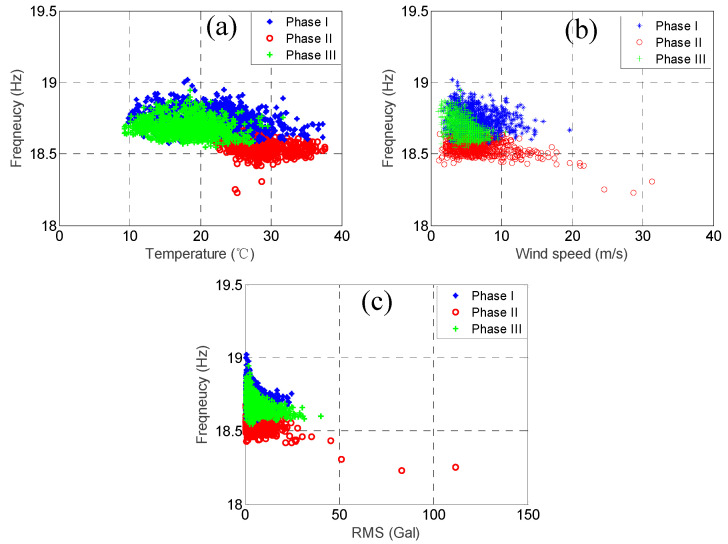
The relationship between the 7th modal frequency (18.67Hz) and (**a**) temperature; (**b**) wind speed; and (**c**) RMS acceleration.

**Figure 18:**
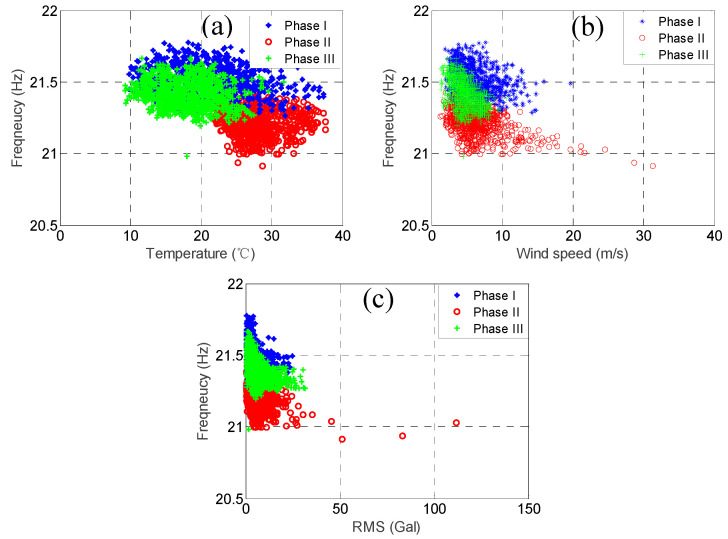
The relationship between the 8th modal frequency (21.39Hz) and (**a**) temperature; (**b**) wind speed; and (**c**) RMS acceleration.

As mentioned previously, the modal frequencies of some of the higher modes but not the lower modes dropped substantially because of Typhoon Nesat. In order to observe this phenomenon more clearly, we plotted the relationship between the three environmental factors and the modal frequencies for the two modes with the highest correlation coefficients of temperature, i.e., the 3rd mode and the 4th mode, and the two modes with the lowest correlation coefficients of temperature, i.e., the 7th mode and the 8th mode, as shown in Figure 15, Figure 16, Figure 17 and Figure 18. In each figure, the data within the different phases are marked using different shapes and colours.

Let us focus on the 3rd mode and the 4th mode first. Figure 15 and Figure 16 show the temperature, RMS acceleration, and wind speed effects on these two modes. The effects exerted on these two modes were evidently dominated by the temperature because their correlation coefficients were much higher than those for the other two environmental factors. It can be observed in Figure 15a and Figure 16a that the temperature effects were quite linear, and the slopes also declined for all the three phases. Note that the temperature ranges of the three phases were quite different. The temperature range of the first phase was relatively wide, ranging from 9.8 °C to 37.2 °C, with the average equal to 23.6 °C. The temperature of the second phase was relatively high, ranging from 21.8 °C to 37.6 °C, with the average equal to 28.4 °C. The temperature of the third phase was relatively low, ranging from 8.9 °C to 31.9 °C, with the average equal to 18.5 °C. In contrast, Figure 15b and Figure 16b show that the wind speed effects were quite ambiguous, consistent with the low wind speed correlation coefficients, i.e., only −0.03 and −0.16, respectively. Although the ranges of the wind speed values during these three phases were not the same, most of their values fell below 10 m/s. Therefore, because the effects exerted on these modal frequencies were dominated by temperature, and the temperature ranges of the three phases were different, the distributions of these three phases in Figure 15b and Figure 16b are also different. For instance, the modal frequencies during the second phase were smaller than the ones during the other two phases because the temperature was relatively high during that phase. As for the RMS acceleration, Figure 15c and Figure 16c show that the effects were also quite small, which again was consistent with the low RMS acceleration correlation coefficients, i.e., only 0.00 and 0.28, respectively. Although their effects were somewhat linear, the slopes were very small. Note that for all three phases, the distributions of the RMS acceleration values were quite similar. Again, due to the dominant temperature effects and the distinct temperature patterns of these three phases, the distributions of these three phases in Figure 15c and Figure 16c are also different. Similar conclusions can be drawn observing the remaining four lower modes.

Let us focus on the 7th mode and the 8th mode now. Figure 17 and Figure 18 show the temperature, RMS acceleration, and wind speed effects on these two modes. Unlike the 3rd mode and the 4th mode, the effects exerted on these two modes were not dominated by any of the three environmental factors, but the effects of the RMS acceleration and wind speed were higher than those of the temperature. Figure 17c and Figure 18c show that the modal frequencies decreased when the RMS acceleration increased, and the relationship was not simply linear, but somewhat convex. These two figures correspond to the RMS acceleration correlation coefficients of these two modes, which are −0.35 and −0.41, respectively, quite well. Since the ranges of the RMS acceleration values during these three phases were quite similar, and the RMS acceleration correlation coefficients were the highest among the three environmental factors, the patterns of the data during these three phases should be quite similar. However, their patterns were evidently different. In general, the modal frequencies during the first phase were the highest, followed by the ones during the third phase, while the ones during the second phase were the lowest. This phenomenon implies that there should have been other effects that caused such remarkable variation in the modal frequencies. Similarly, Figure 17b and Figure 18b show that the modal frequencies were also decreased when the wind speed was increased, but the relationship was not as convex as that for the RMS acceleration. These two figures also correspond to the wind speed correlation coefficients of these two modes, which are −0.25 and −0.35, respectively, quite well. Knowing that the RMS acceleration values during these three phases were quite similar, and that most of the wind speed values of the three phases were below 10 m/s, it can be concluded that the patterns of data during these three phases should be quite similar if we already know that the modal frequencies of the two modes were mainly affected by the RMS acceleration and wind speed but not the temperature. However, their patterns were evidently different, and the trends were similar to the ones shown in Figure 17c and Figure 18c. Again, this phenomenon implies that there should have been other effects that caused such remarkable variation in the modal frequencies. As for the temperature, Figure 17a and Figure 18a show that the modal frequencies were also decreased when the temperature was increased, but the relationships were quite linear and the slopes were not so inclined. The temperature correlation coefficients of these two modes were only −0.17 and −0.14, respectively. However, as already mentioned previously, while the temperature range of the three phases were quite different, since their temperature effects were not large, the remarkable variation in the modal frequencies should not be attributed to the temperature variation. As a result, the remarkable variation in the modal frequencies cannot have been due to any of the three environmental effects. Hence, it is very possible that they were due to the Typhoon Nesat event. Similar conclusions can be drawn observing the remaining two higher modes.

Note that for these two typical higher modes, during these three phases, the RMS acceleration and wind speed were generally under similar conditions, and the temperature effects were not large. Therefore, the change in the variation percentage intercepts of the linear equations of the three phases was considered to have been affected by the Typhoon Nesat event and also the recovery mechanism due to the seasonal temperature change. As shown in Figure 3, unlike other civil engineering structures whose dominant vibration signal is mainly contributed by the lower modes, the dominant vibration signal of the PTT is mainly contributed by the vibration frequencies close to 25 Hz. This finding supports the argument that it was more likely that the modal frequencies of the higher modes were altered by the Typhoon Nesat event.

Because among the three environmental factors, the relationship between the temperature and the modal frequency was the most linear and simplest, a simple first-degree polynomial equation was used to fit the data of the three different phases individually using the least squares method. The change in the intercept of the linear equations for each modal frequency was calculated using the first phase as a reference, with the results listed in Table 3. It can be observed that for the second phase, the variation percentage of the first six modal frequencies was relatively small, approximately between −0.07% and −0.46%, while the variation percentage of the remaining four higher modes was relatively large, approximately between −0.89% and −1.34%. For the third phase, the values of the variation percentage of all the modes were increased. For the six lowest modes, the variation percentage was between −0.19% and 0.26%, while for the four highest modes, the variation percentage was between −0.45% and 0.68%. The largest change in the variation percentage from the second phase to the third phase was in the 9th mode, for which it was increased from −1.34% to 0.68%, a difference of approximately 2%.

## 6. Conclusions and Discussion

In this study, the acceleration time history, temperature, and wind speed recorded every hour by the long-term monitoring system of a PTT were analysed. The automated SSI technique was employed to identify the candidate modes of the PTT using the acceleration time history of each dataset recorded every hour. These candidate modes were traced using a proposed procedure with four steps, and the ones with relatively high identifiability were selected as the target modes to be investigated for their environmental effects. Not only the lower modes, but also the higher modes were selected. Note that because the PTT is a very complex truss structure, it is not easy to interpret the identified modes using only a few DOFs of acceleration measurement in this study. Nevertheless, the modes with different frequencies and mode shapes were identified successfully using such small number of accelerometers.

The ten months of monitoring data were adopted to examine the variation trends of the ten target modal frequencies and assess the effects of the temperature, vibration intensity, and wind speed. In general, when the vibration intensity was larger, the identifiability of the modal frequencies for that month was also larger. The variation range of all the target modes was not very large, only between 2% and 8%. For all the target modes, the modal frequencies decreased as the temperature increased, with a nearly linear relationship. However, the correlation coefficients were somewhat diverse, ranging from −0.14 to −0.90. As for the wind speed, all the modal frequencies of the target modes decreased as the wind speed increased, but with smaller amplitudes of correlation coefficients, which ranged from −0.03 to −0.40. As for the RMS acceleration, for the five lower target modes, their correlation coefficients were positive, ranging from 0.00 to 0.34, whereas for the five higher target modes, their correlation coefficients were negative, ranging from −0.16 to −0.41. The relationships between the modal frequencies and both the RMS acceleration and wind speed were somewhat convex, but not clear.

During the whole monitoring period of 2017, the Typhoon Nesat event affected the PTT on 29 July, and seems to have temporarily affected the values of the modal frequencies, especially those for the higher target modes. This temporary effect of the typhoon event seems to have been recovered from when the temperature started to cool down beginning around 14 October. Accordingly, the long-term monitoring period was divided into three phases to be investigated for the typhoon and environmental effects. After deliberately examining the modal frequencies of these three phases together with the environmental factors of the three phases, it was observed that the modal frequencies dropped substantially after the typhoon event, especially for the higher modes, except the 5th mode, whose modal frequencies dropped by approximately −0.9% to −1.35%. As for the remaining lower modes, although their modal frequencies also dropped after the typhoon event, the amplitudes of these drops were observed to be approximately only −0.14% to −0.35%. It is possible that the sudden drop of natural frequencies is due to the significant amount of precipitation that usually occurs during typhoon events. Precipitation in the form of rain adds to the inertia of the structure, and in addition, it also affects the soil where the structure’s foundation is placed. Increased inertia and softened, moist soil could be the factors causing a reduction in the natural frequencies. Unfortunately, the instrumentation of the PTT did not include a precipitation sensor and the humidity sensor, so it will not be possible to check if these factors have a significant correlation with natural frequencies. Nevertheless, it remains unclear why only the higher modes were affected significantly, hence further studies are required to understand this phenomenon.

In this study, environmental and typhoon effects on a PTT were explored and quantified. The next step would be to eliminate those interferences from the variations of the target modal frequencies as much as possible. It can be imagined that such work would face challenges because of the diverse phenomena associated with the effects of temperature, RMS acceleration, wind speed, and typhoon events. Nonetheless, it is hoped that in the near future, a damage detection approach using the measured modal frequencies will be applied successfully based on the results in the current study.

## Figures and Tables

**Table 1:** Basic information of the ten target modes.

Mode No.	Identifiability (%)	Average Frequency (Hz)	Variation Range (Hz)	Variation Percentage (%)	Category
1	95.22	2.03	0.12	5.78	Bending
2	96.06	2.43	0.06	2.63	Bending
3	97.31	2.51	0.05	2.18	Bending
4	94.58	3.26	0.16	4.86	Bending
5	80.68	8.26	0.46	5.56	Bending
6	61.83	15.33	0.50	3.23	Torsion
7	80.34	18.67	0.79	4.24	Torsion
8	71.63	21.39	0.87	4.05	Bending
9	71.33	24.36	1.92	7.89	Local
10	61.75	31.08	1.68	5.39	Torsion

**Table 2:** Pearson correlation coefficients for the three environmental factors and the target modal frequencies during the first phase.

Mode No.	Temperature	Wind Speed	RMS Acceleration
1	−0.43	−0.08	0.34
2	−0.51	−0.09	0.00
3	−0.77	−0.03	0.28
4	−0.90	−0.16	0.20
5	−0.61	−0.18	0.15
6	−0.60	−0.40	−0.16
7	−0.17	−0.25	−0.35
8	−0.14	−0.35	−0.41
9	−0.52	−0.32	−0.29
10	−0.35	−0.29	−0.18

**Table 3:** The change of intercept of the first-degree polynomial equations for each modal frequency using the first phase as reference.

Mode No.	Change of Intercept			
	Phase II		Phase III	
	(Hz)	(%)	(Hz)	(%)
1	−0.003	−0.14%	−0.002	−0.09%
2	−0.009	−0.35%	−0.005	−0.19%
3	−0.005	−0.18%	−0.003	−0.10%
4	−0.011	−0.33%	−0.006	−0.18%
5	−0.038	−0.46%	−0.011	−0.14%
6	−0.010	−0.07%	0.040	0.26%
7	−0.166	−0.89%	−0.066	−0.35%
8	−0.266	−1.24%	−0.096	−0.45%
9	−0.332	−1.34%	0.168	0.68%
10	−0.307	−0.98%	0.003	0.01%
